# Plug-assisted retrograde transvenous obliteration via gastrocaval shunt for the gastric variceal bleeding: A case report

**DOI:** 10.1097/MD.0000000000028107

**Published:** 2021-12-10

**Authors:** Joo Yeon Jang, Ung Bae Jeon, Jin Hyeok Kim, Tae Un Kim, Hwaseong Ryu, Mong Cho, Young Mi Hong, Ki Tae Yoon

**Affiliations:** a Department of Radiology, Pusan National University Yangsan Hospital, College of Medicine, Pusan National University, Busan, Korea; b Department of Internal Medicine, Pusan National University Yangsan Hospital, College of Medicine, Pusan National University, Busan, Korea.

**Keywords:** case report, gastric varix, gastrocaval shunt, liver cirrhosis, plug-assisted retrograde transvenous obliteration

## Abstract

**Rationale:**

Most gastric varices at the fundus drain into the left renal vein via the gastrorenal shunt (80–85% of cases) or the inferior vena cava via the gastrocaval shunt (10–15%). Therefore, plug-assisted retrograde transvenous obliteration (PARTO) is usually performed via a gastrorenal shunt. Here, we report a case of gastric varix treated with PARTO via a gastrocaval shunt.

**Patient concerns:**

A 46-year-old woman with hepatitis B virus and liver cirrhosis visited the emergency room in our hospital with the main symptom of hematemesis and hematochezia.

**Diagnoses:**

Endoscopy and computed tomography (CT) revealed a gastric varix and thrombotic-occluded transjugular intrahepatic portosystemic shunt (TIPS) stent.

**Interventions:**

The patient underwent PARTO via a gastrocaval shunt to manage gastric variceal bleeding after failed TIPS revision.

**Outcomes:**

On CT, the gastric varix completely disappeared. The patient did not experience any additional bleeding events.

**Lessons:**

PARTO via a gastrocaval shunt is safe and effective.

## 1 Introduction

Gastric variceal bleeding is a significant complication of portal hypertension in patients with cirrhosis and has a poor prognosis and a high mortality rate.^[1–4]^ Anatomically, the majority of gastric varices located at the fundus drain into the inferior phrenic vein, which later joins with the left renal vein via the gastrorenal shunt (80–85%) or with the inferior vena cava (IVC) via the gastrocaval shunt (10–15%).^[5,6]^ Balloon-occluded retrograde transvenous obliteration (BRTO), with the concept of retrograde injection of a sclerosing agent into the gastric varix after balloon occlusion of the gastrorenal shunt, has become the method of choice for the control of fundal gastric varices with such anatomy.^[7]^

Recently, plug-assisted retrograde transvenous obliteration (PARTO) was reported, in which an Amplatzer vascular plug was used instead of a balloon.^[8]^ It has been more popular than the previous BRTO procedure because of its shorter procedure time and better efficacy. To our knowledge, there are no reports on PARTO performed with other shunts, such as a gastrocaval shunt. We report a case of gastric varix treated with PARTO via a gastrocaval shunt without a gastrorenal shunt.

## 2 Case report

A 46-year-old woman presented to the emergency room with hematemesis and hematochezia. She had been diagnosed with hepatitis B and liver cirrhosis 11 years previously. She had undergone a transjugular intrahepatic portosystemic shunt (TIPS) procedure for gastroesophageal varix bleeding 3 years earlier. The initial blood pressure was 130/50 mm Hg, but dropped to 80/60 mm Hg after 3 hours. Laboratory values were as follows: hemoglobin 10.2 g/dL; platelet count 72 × 103/μL; serum bilirubin 1.3 mg/dL; albumin 3.5 g/dL; prothrombin time/international normalized ratio 1.48; aspartate aminotransferase 38 IU/L; and alanine aminotransferase 23 IU/L. At that time, there was no evidence of hepatic encephalopathy or ascites. Therefore, her Child-Pugh score was class A at 6 points.

**Figure 1:**
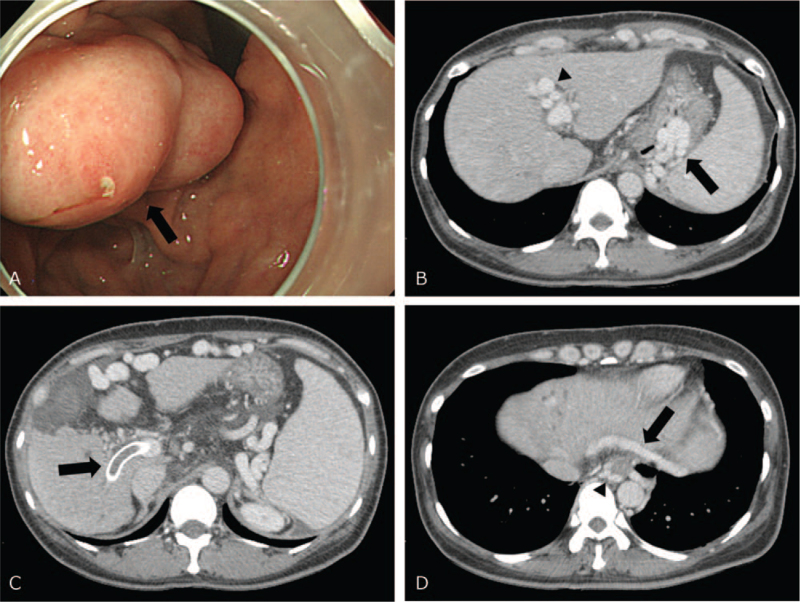
(A) Endoscopic image shows a large polypoid mass with stigmata (arrow) without active bleeding on the stomach. (B) CT shows underlying liver cirrhosis with splenomegaly, umbilical varix (arrowhead), and gastric varix (arrow). (C) A previously inserted TIPS stent (arrow) is occluded by a thrombus. (D) Only gastrocaval shunt (arrow) and esophageal varix (arrowhead) are seen.

Endoscopy showed scar change and tortuous venous dilatation without stigmata on the esophagus, but a sizeable polypoid mass with stigmata without bleeding on the stomach fundus (Fig. 1A). Computed tomography (CT) showed liver cirrhosis with splenomegaly, umbilical varix, and gastroesophageal varices (Fig. 1B). In addition, the previously inserted TIPS stent was occluded by a thrombus (Fig. 1C). TIPS revision was initially considered for gastric varix.

The patient was transferred to the angiography suite. The right internal jugular vein was punctured under ultrasound guidance. Subsequently, a 10 Fr sheath (Flexor Check-Flo II Introducer Set, Cook, Bloomington, IN) was inserted. The right hepatic vein was selected to approach the TIPS stent. Then, TIPS stent passage was attempted using a 0.035-inch, 150-cm-long hydrophilic guidewire (Terumo, Tokyo, Japan), a 5 Fr Cobra catheter (Merit Medical, South Jordan, UT), and a 5 Fr Davis catheter (A&A Medical Device, Seongnam, South Korea). However, it failed because of severe obstruction of the stent.

**Figure 2:**
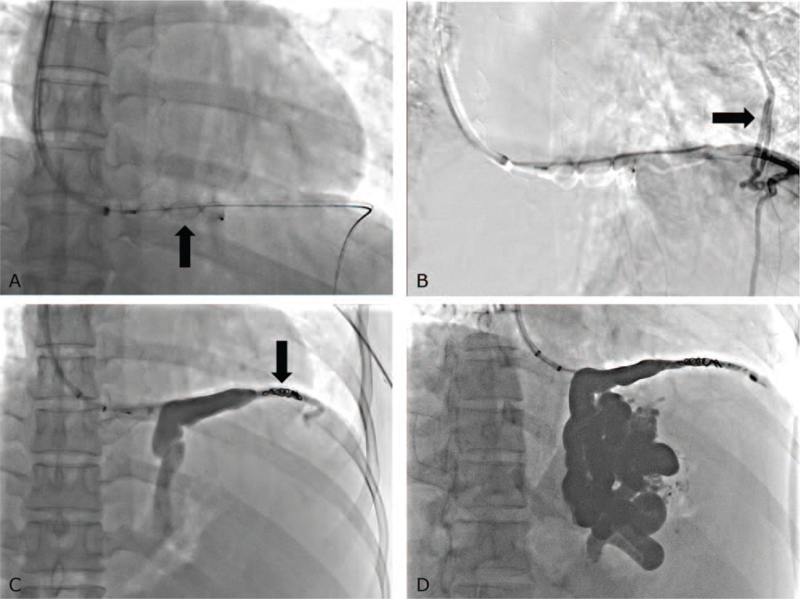
(A) 12 mm Amplatzer Vascular Plug II (arrow) is inserted into the gastrocaval shunt. (B) Venography using a 4 Fr Cobra catheter shows no visualization of gastric varix due to large collateral vessels, such as the pericardiophrenic vein (arrow). The catheter is placed just proximal to the plug. (C) After embolization of collateral vessels using coils (arrow), the gastrocaval shunt and gastric varix can be visualized. (D) The gastrocaval shunt, gastric varix, and afferent vein are full of gelatin sponges at the end of the procedure.

In this patient, there was no gastrorenal shunt, but a gastrocaval shunt (Fig. 1D). Therefore, classical PARTO via a gastrorenal shunt was not possible, but a gastrocaval shunt could be an alternative to gastric varix. After the left inferior phrenic vein (transverse portion) negotiation through the IVC, the 7F Ansel sheath (Cook) was inserted into the gastrocaval shunt. A 12 mm Amplatzer Vascular Plug II (Abbott Medical, Plymouth, MN) was placed in the gastrocaval shunt (Fig. 2A). Venography showed no visualization of gastric varix due to large collateral vessels, such as the pericardiophrenic vein, after placing 4 Fr Cobra catheter (Terumo, Tokyo, Japan) into the gastrocaval shunt proximal to the vascular plug (Fig. 2B). The collateral vessel was embolized using coils (6 mm 035 Nester coil, Cook) (Fig. 2C). Gelatin sponges (Cutanplast; Mascia Brunelli Spa, Milano, Italy) were injected through the catheter toward the gastrocaval shunt and gastric varix until the afferent veins were sufficiently opacified (Fig. 2D). After embolization, the catheter was removed, and the vascular plug was detached.

During the procedure, the patient's vital signs were stable. The day after the procedure, she experienced additional melena. After that, there was no bleeding event; therefore, she was discharged. Approximately 2 weeks after the procedure, laboratory findings showed hemoglobin 10.1 g/dL, platelet count 80 × 103/μL, serum bilirubin 1.0 mg/dL, albumin 3.5 g/dL, aspartate aminotransferase 27 IU/L, and alanine aminotransferase 16 IU/L.

**Figure 3:**
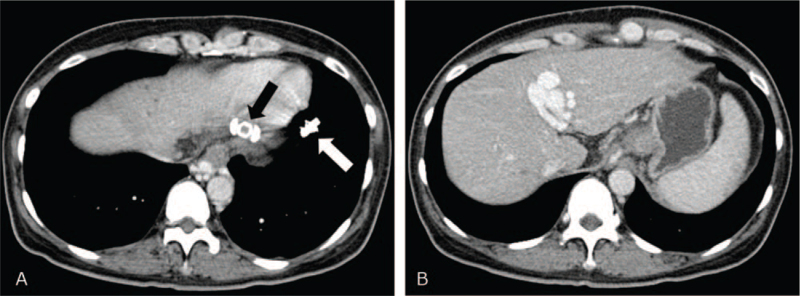
(A) Approximately 9 months later, Amplatzer vascular plug (arrow) and coils (white arrow) are seen in embolized gastrocaval shunt and collaterals. (B) Gastric varix completely disappears.

Follow-up CT after 9 months showed an Amplatzer vascular plug, coils in the embolized gastrocaval shunt, and collateral veins, and disappearance of gastric varix (Fig. 3). Until now, the esophageal varix has been stable, and the patient has presented no episode of bleeding.

## 3 Discussion

In patients with liver cirrhosis, gastroesophageal variceal bleeding is a significant complication associated with portal hypertension.^[1]^ Although bleeding from gastric varices is relatively low (10–36%) compared with esophageal varices, it results in a high mortality rate (14–45%).^[2,3,9,10]^ Therefore, various treatment methods have been developed to manage gastric varix, including endoscopic injection sclerotherapy, percutaneous transhepatic obliteration, TIPS, and BRTO. BRTO has been widely used as a highly effective and minimally invasive treatment method for gastric varix since its introduction by Kanagawa et al^[7]^ in 1996.

Gastric varices can be formed from several gastric veins, including the left gastric vein, posterior gastric vein, and short gastric vein. However, most GVs flow into the left renal vein via a gastrorenal shunt. From this perspective, the idea of retrograde injection of sclerosing agents into the GV after gastrorenal shunt balloon occlusion has been developed.^[7]^ Ethanolamine oleate is the most commonly used sclerosant in BRTO. Therefore, various complications associated with ethanolamine oleate and balloon rupture have been reported.

PARTO was introduced by Gwon et al^[8]^ in 2013. In PARTO, a vascular plug and gelatin sponge were used instead of a balloon catheter and ethanolamine oleate, respectively. Therefore, PARTO is technically feasible, safe, and time-efficient. As a result, PARTOs are commonly used worldwide.

As mentioned earlier, a gastrorenal shunt is the primary drainage route for gastric varices. Therefore, almost all PARTO procedures are performed via a gastrorenal shunt. However, some patients do not have gastrorenal shunts, but other channels, such as our patient. Only 10% to 15% of gastric varices drain into the IVC via the gastrocaval shunt. The left inferior phrenic vein passes below the diaphragm transversely in front of the esophagus to form a gastrocaval shunt and terminates in the IVC (60%) or the left hepatic vein (40%). Therefore, the proximal portion of the shunt runs under the diaphragm; however, the distal branches run superiorly to the diaphragm and connect with other peridiaphragmatic veins; thus, gastrocaval shunts always communicate with multiple accessory drainage veins around the diaphragm.^[11]^

Some reports have described BRTO via a gastrocaval shunt using balloon catheters.^[6]^ Transfemoral or transjugular approaches were chosen to approach the gastrocaval shunt to the gastric varix with microballoon catheters to inject sclerosing agents such as ethanolamine oleate. PARTO is based on a similar concept for treating gastric varices. It can be performed via a gastrocaval shunt using an Amplatzer plug and gelatin sponges instead of balloon catheters and sclerosing agents.

In conclusion, PARTO can also be successfully performed in a patient with a gastrocaval shunt. The operator should be aware of the shunt pathway to the gastric varix on CT before performing PARTO for gastric varix treatment.

## Author contributions

**Conceptualization:** Joo Yeon Jang, Jin Hyeok Kim.

**Formal analysis:** Ung Bae Jeon, Tae Un Kim, Hwaseong Ryu.

**Funding acquisition:** Joo Yeon Jang, Ung Bae Jeon

**Resources:** Joo Yeon Jang, Mong Cho.

**Supervision:** Ung Bae Jeon.

**Visualization:** Young Mi Hong, Ki Tae Yoon

**Writing – original draft:** Joo Yeon Jang

**Writing – review & editing:** Joo Yeon Jang, Ungbae Jeon, Jin Hyeok Kim, Tae Un Kim, Hwaseong Ryu, Mong Cho, Young Mi Hong, Ki Tae Yoon.
